# Correlations between MSH2 and MSH6 Concentrations in Different Biological Fluids and Clinicopathological Features in Colorectal Adenocarcinoma Patients and Their Contribution to Fast and Early Diagnosis of Colorectal Adenocarcinoma

**DOI:** 10.3390/biomedicines11123213

**Published:** 2023-12-04

**Authors:** Alexandru Adrian Bratei, Raluca-Ioana Stefan-van Staden

**Affiliations:** 1 Faculty of Chemical Engineering and Biotechnologies, University Politehnica of Bucharest, 060042 Bucharest, Romania; brateialexandru@yahoo.com; 2 Laboratory of Electrochemistry and PATLAB, National Institute of Research for Electrochemistry and Condensed Matter, 060021 Bucharest, Romania; 3 Department of Pathology, George Emil Palade University of Medicine, Pharmacy, Science and Technology of Targu-Mures, 540139 Targu-Mures, Romania

**Keywords:** colorectal adenocarcinoma, MSH2, MSH6, clinicopathological features, stochastic sensor, bioanalysis

## Abstract

(1) Background: The human MutS homolog, hMSH2, is known to be involved in DNA mismatch repair and is responsible for maintaining the stability of the genome. When DNA damage occurs, MSH2 promotes cell apoptosis via the regulation of ATR/Chk2/p53 signal transduction, and MSH2 deficiency is also related to accelerated telomere shortening in humans. MSH2 missense mutations are involved in a defective DNA reparation process, and it can be implied in carcinogenesis, as it is already involved in well-known cancer-related syndromes such as Lynch syndrome. Human MSH6, which stands for mutS homolog 6, is a member of the MMR family that is responsible for the repair of post-replicative mismatched DNA bases. It is also one of the proteins with gene mutations that are associated with a high risk of developing Lynch syndrome, leading to a large series of tumors. (2) Methods: Patients and their clinical and pathological features were selected from the database of the project GRAPHSENSGASTROINTES and used accordingly, with ethics committee approval no. 32647/2018 awarded by the County Emergency Hospital from Targu-Mures. Analyses were conducted on whole blood, saliva, urine, and tumoral tissue samples using a stochastic method with stochastic microsensors. (3) Results: The results obtained using stochastic sensors were correlated with a series of macroscopic and microscopic pathological features for each sample type. Criteria or relationships were established for tumor location, vascular and perineural invasions, lymph node metastases, the presence of tumor deposits, and the presence of a mucus compound in the tumor mass. (4) Conclusions: The correlation between the concentrations of MSH2 in the four types of samples and the pathological features allowed for the fast characterization of a tumor, which can help surgeons and oncologists choose personalized treatments. Also, the colorectal tumor location was correlated with the concentration of MSH2 in whole blood, urine, and saliva. MSH6, which stands for mutS homolog 6, is not only useful in immunohistochemistry but in pathology practice as well. In this paper, the relationships between MSH6 levels in four biological fluids—whole blood, saliva, urine, and tissues—and tumor locations among the colorectal area, gross features, presence of a mucinous compound, molecular subtype, stroma features, and vascular invasions are presented.

## 1. Introduction

It is well-known that cells develop mechanisms that ensure high-fidelity transmission of genetic material over generations since mutations can induce a series of disturbances to the cell. DNA lesions related to mutations are missing, modified, or mismatched nucleotides [1] that are repaired through multiple enzymatic pathways [2]. One of these pathways is the *Escherichia coli* MutHLS pathway, which associates an excision repair reaction using MutH, MutL, and MutS proteins [3,4]. During replication, MutS is responsible for binding to mismatched nucleotides in DNA [5]; this process is facilitated by MutL [6]. Biochemical studies have revealed that eukaryotes have similar repair systems [7,8], and the human MutS homolog, hMSH2, is highly structurally related to the MutS homolog in *S. cerevisiae* [9]. Moreover, MSH2 was shown to be involved in DNA mismatch repair (MMR) [10,11], which is responsible for maintaining the stability of the genome.

Biochemical analyses have shown that the MSH2 protein is composed of 934 amino acidic residues, and, in vivo, hMSH2 and hMSH6 proteins form a heterologous heterodimer (hMutSα), which recognizes and binds to the mismatch site [12,13]. Moreover, hMutSα and hMutL heterodimers (composed of hMLH1 and hPMS2) constitute a polymer responsible for the recruitment of other proteins needed for mismatch repair [14,15]. The two proteins, MSH2 and MSH6, can also form a complex with BLM-p53-RAD51 as part of the DNA damage repair process [16], and when DNA damage occurs, MSH2 promotes cell apoptosis by regulating ATR/Chk2/p53 signal transduction [17]. In addition, MSH2 deficiency is related to accelerated telomere shortening in humans [18,19]. MSH2 missense mutations are involved in a defective DNA reparation process, and it can be implied in carcinogenesis [20]. Recently, it was shown that MSH2 is linked to the occurrence of Lynch syndrome, which is an autosomal dominant genetic disease associated with a high risk of developing colorectal cancer and many others [21,22,23].

Human MSH6 (mutS homolog 6) is a member of the MMR’s MutS family, which is also known as p160 or GTBP, and it is responsible for post-replicative mismatched DNA base repair [24,25]. Its gene is primarily located on chromosome 2′ short arm, and, as with all MutS homologs, it contains a Walker-A/B adenine nucleotide motif related to intrinsic ATPase activity [26]. During the reparation process, it binds with MSH2, forming a heterodimer complex that recognizes mismatches [27,28]. 

Biochemically, the MSH6 protein structure is comparable to that of *E. coli* MutS, as it contains five conserved domains and a disordered N-terminal PWWP domain [12,29,30]. Mutations lead to differences in these domains and are related to tumorigenesis in a series of cancers [31,32,33,34]. 

Carriers of the four MMR gene mutations present a high risk of developing Lynch syndrome [35,36,37,38], which is associated with cancers in different locations, such as in the colorectum, stomach, ovary, biliary tract, and skin, and it requires germline genetic testing for identifying high-risk individuals [39,40]. MSH6 mutations account for 10–20% of Lynch syndrome cases and, for these patients, colonoscopy should begin at 30 years and be repeated every 1–2 years [41,42,43]. 

In this paper, the levels of MSH2 and MSH6 were evaluated in whole blood, saliva, urine, and tissue samples, and they are presented and discussed in addition to correlations with clinicopathological features.

## 2. Materials and Methods

### 2.1. Patient Description

After receiving informed consent, four kinds of samples from 116 colorectal cancer patients (107 whole blood samples, 79 saliva samples, 88 urine samples, and 60 tissue samples) were collected. The patients were selected from the GRAPHSENSGASTROINTES project database, and their data were used according to the ethics committee’s approval (No. 32647/2018), which was granted by the County Emergency Hospital from Targu-Mures. Among the selected patients, immunohistochemical tests were conducted only on 93 patients for MSH2 mutations and only on 90 patients for MSH6 mutations. All the tested samples were associated with a positive stain reaction.

### 2.2. Materials and Reagents

All chemicals used were of analytical grade. Biomarker extracts were purchased from Sigma Aldrich, and paraffin oil (d_4_^20^, 0.86 g/cm^3^) was purchased from Fluka, Germany. The stochastic sensors were designed and characterized as described earlier.

### 2.3. Instrumentation

All measurements were performed using an Autolab PGSTAT 302 (Metrohm, Utrecht, The Netherlands) connected to a computer equipped with GPES software. The electrochemical cell included a stochastic microsensor, a reference electrode (Ag/AgCl), and an auxiliary electrode (Pt). 

### 2.4. Stochastic Method

A stochastic microsensor based on nitrogen (9.3%) and boron (2.4%)-doped graphene (NB-DG) modified with frutafit HD and integrated into a screening platform was used as a screening tool for the biological samples [22]. The chronoamperometric method was used for the molecular recognition and quantification of MSH2 and MSH6 in biological samples. The stochastic method is based on channel conductivity: when a molecule enters the channel, the current drops to zero until the molecule enters the channel (the time needed for the molecule to enter the channels is the signature of the analyte, and is referred to as *t*_off_); while in the channel, the molecule undergoes redox processes for a certain time—*t*_on_—and this value (measured in between two *t*_off_ values) is correlated with the concentration of the biomarkers [22]. The molecular recognition was performed based on their signature (the *t*_off_ value), which was 1.4 s for MSH2 and 2.3 s for MSH6. Their quantification was made using the *t*_on_(s) values, and the following equations for calibration: for MSH2: 1/*t*_on_ = 0.18 + 5.31 × C and for MSH6: 1/*t*_on_ = 0.07 + 1.58 × 10^3^ × C (<C> = μg mL^−1^) [22]. A constant potential of 125 mV was applied for the simultaneous determination of MSH2 and MSH6 in the biological samples. The designed microsensors were introduced into a cell containing the biological samples, the potential was applied, and the diagrams were obtained for each of the biological samples.

## 3. Results

The concentrations of MSH2 and MSH6 were determined and measured in all four biological fluids (tissue, whole blood, saliva, and urine). In order to use them in medical practice, a series of cut-off values were established for both biomarkers. The cut-off values were chosen empirically based on mathematical techniques in order to maximize the separation grade of patients for each clinicopathological feature.

Before determining the cut-off values, some parameters, which are important for establishing the location, were defined:

1. r_1_ = [X]_whole blood_/[X]_urine_.
2. r_2_ = [X]_whole blood_/[X]_saliva_.
3. S = [X]_saliva_ + 2 × [X]_urine_.

where [X] represents the concentration (pg/mL) of the biomarker X.

The colorectum was divided into six areas—ascending colon (C1), transverse colon (C2), descending colon (C3), sigmoid colon(C4), rectosigmoid junction(C5) and rectum(C6). For cancers located in these areas, a series of criteria that can statistically predict if a tumor is located there were established. These criteria are detailed in the Section 4.

The cut-off values for MSH2 are given in Table 1.

The values in Table 1 were established by analyzing information from the database from County Emergency Hospital of Targu-Mures, and the values were obtained using a stochastic method. Regarding the cut-off values and their applicability, a series of observations were made:

- C1-located tumors are associated with r_1_ > 1.5, S > 500 pg/mL, r_2_ < 0.75, and [MSH2]_whole blood_ > 50 pg/mL;
- C2 + C3-located tumors are associated with r_1_ < 1.5, S > 500 pg/mL, r_2_ > 0.75, and [MSH2]_whole blood_ > 50 pg/mL;
- C4-located tumors are associated with r_1_ < 1.5, S < 500 pg/mL, r_2_ < 0.75, and [MSH2]_whole blood_ > 50 pg/mL;
- C5-located tumors are associated with r_1_ < 1.5, S < 500 pg/mL, r_2_ < 0.75, and [MSH2]_whole blood_ < 50 pg/mL;
- C6-located tumors are associated with r_1_ < 1.5, S > 500 pg/mL, r_2_ < 0.75, and [MSH2]_whole blood_ > 50 pg/mL;
- Vegetant gross aspect is associated with [MSH2]_whole blood_ < 150 pg/mL;
- Mucus presence is associated with [MSH2]_whole blood_ < 125 pg/mL and [MSH2]_saliva_ < 140 pg/mL;
- Blood vessel invasion is associated with [MSH2]_whole blood_ < 160 pg/mL;
- Lymph vessel invasion is associated with [MSH2]_whole blood_ < 125 pg/mL;
- Tumor deposit presence is associated with [MSH2]_saliva_ < 125 pg/mL, [MSH2]_whole blood_ < 125 pg/mL, and [MSH2]_urine_ < 150 pg/mL.

A similar approach was made for MSH6, and the selected cut-off values are given in Table 2.

Some observations regarding the cut-off values given in Table 2 include:

- C2 + C3-located tumors are associated with [MSH6]_urine_ > 250 pg/mL, S > 600 pg/mL, r_2_ = 0.3–1.2, and [MSH6]_whole blood_ = 130–300 pg/mL;
- C5-located tumors are associated with r_1_ < 0.5, S > 1500 pg/mL, r_2_ < 0.83, and [MSH6]_whole blood_ > 130 pg/mL;
- C1 + C4 + C6 cannot be differentiated with high probability using only MSH6 levels;
- Vegetant and ulcero-vegetant gross aspect is associated with [MSH6]_urine_ < 220 pg/mL;
- Mucus presence is associated with [MSH6]_tissue_ > 250 pg/mL and [MSH6]_saliva_ > 250 pg/mL;
- The presence of an epithelial subtype compound is related to [MSH6]_urine_ < 200 pg/mL;
- Stroma features are related to urine MSH6 levels as [MSH6]_urine_ > 300 pg/mL involves a hyalinized stroma and [MSH6]_urine_ < 250 pg/mL involves mainly inflammatory stroma;
- Blood vessel invasion is associated with [MSH6]_urine_ > 450 pg/mL.

## 4. Discussion

Nowadays, most uses of MSH2 and MSH6 in pathology are based on immunohistochemical stain reactions. Significant correlations with cancer prediction, prognosis [44], clinicopathological features [45], associated mutations, and treatment response [46] have been reported recently. In our paper, we present the results obtained with a complementary method to classical immunohistochemistry. While immunohistochemistry checks the biomarkers’ presence in tumor tissue, the measurements with stochastic sensors evaluate the release of biomarkers in extracellular fluids, giving supplementary information about the tumor.

### 4.1. MSH2 and Its Clinicopathological Features

The results obtained using stochastic methods proved to be extremely useful for a clinicopathological characterization of the colorectal cancer patients. The features collected in the database were analyzed relative to the MSH2 concentration in each biological fluid in order to evaluate any correlation. 

Firstly, the relationship between MSH2 levels in three biological fluids (whole blood, urine, and saliva) was analyzed. Only these three fluids were used in order to establish the location because the aim was to locate the tumor to obtain a tissue sample, so tissue sample MSH2 concentration would not be useful.

Four criteria with clinical significance were analyzed—the whole blood MSH2 concentration, reflecting the mass transfer from the tumor mass to blood, the ratio between whole blood MSH2 concentration and urine MSH2 concentration, reflecting the elimination of MSH2 via the kidneys, the ratio between whole blood MSH2 concentration and saliva MSH2 concentration, reflecting the elimination of MSH2 via the salivary glands, and the sum S = 2 × [MSH2]_urine_ + [MSH2]_saliva_, reflecting the total elimination via both ways under the assumption that urine daily volume is twice the saliva daily volume.

For these quantifiable parameters, cut-off values were proposed. These values were 50 pg/mL for the whole blood MSH2 level, 1.5 for the ratio of the whole blood MSH2 concentration to urine MSH2 concentration, 0.75 for the ratio of the whole blood MSH2 concentration to urine MSH2 concentration, and 500 pg/mL for the sum S. 

Using these parameters, the locations were divided into three categories—high probability (considered for ≥50% of the patients), medium probability (considered for 25–50% of the patients), and low probability (considered for ≤25% of the patients) —as given in Table 3. In this table, the locations are associated with probability categories in order to determine each location’s characteristics.

As observed in Table 3, some criteria are specific for each location, allowing the distinction between different locations. For these locations, criteria groups were elaborated, for which the percentages of patients who had at least three and at least two out of the four criteria mentioned in each group (given in brackets for each location below) were calculated. 

For the ascending colon, the following were proposed: [MSH2]_whole blood_/[MSH2]_urine_ > 1.5, S > 500 pg/mL, [MSH2]_whole blood_/[MSH2]_saliva_ < 0.75, and [MSH2]_whole blood_ > 50 pg/mL. The percentages for each location were—for C_1_, (71.43%, 100%); for C_2_ + C_3_, (0%, 40%); for C_4_, (18.75%, 43.75%); for C_5_, (0%, 0%); and for C_6_, (20%, 65%). This criteria group can be used to specify, with a high probability, if a tumor is located on the ascending colon in the case that at least three criteria are met. It can exclude C_2_ + C_3_ if at least three criteria are met and C_5_ if at least two criteria are met. When only two criteria are met, supplementary information is needed to determine if the location is on the ascending colon.

For the transverse and descending colons, a second criteria group can be used given by [MSH2]_whole blood_/[MSH2]_urine_ < 1.5, S > 500 pg/mL, [MSH2]_whole blood_/[MSH2]_saliva_ > 0.75, and [MSH2]_whole blood_ > 50 pg/mL. The percentages for each location are—for C_1_, (42.9%, 85.71%); for C_2_ + C_3_, (80%, 100%); for C_4_, (43.75%, 68.75%); for C_5_, (33.3%, 33.3%); and for C_6_, (40%, 75%). Differentiation from C_1_ can be made using the first criteria group, differentiation from C_4_ and C_6_ can be made with data from Table 1 without a possible exclusion of them using only MSH2 levels, and differentiation from C_5_ can be made using [MSH2]_whole blood_/[MSH2]_saliva_ > 0.75 (66.6% vs. 25%) and S > 500 pg/mL (50% vs. 12.5%). C_2_ tumors can be differentiated from C_3_ tumors using saliva MSH2 level, as all the patients with transverse colon-located tumors have values > 100 pg/mL, and all the patients with descending colon-located tumors have values < 100 pg/mL.

All these results can indicate with a high probability the location, but for a probability of nearly 100%, other biomarkers are needed.

Regarding macroscopic features, some observations were also made. In tissue samples, a vegetant gross aspect of adenocarcinomas was slightly related to a lower MSH2 level as 65.4% of the tumors that have a vegetant compound (vegetant or ulcerated and vegetant tumors) are associated with MSH2 levels < 150 pg/mL compared with only 43.3% for the ones that do not have a vegetant compound. 

**Figure 1:**
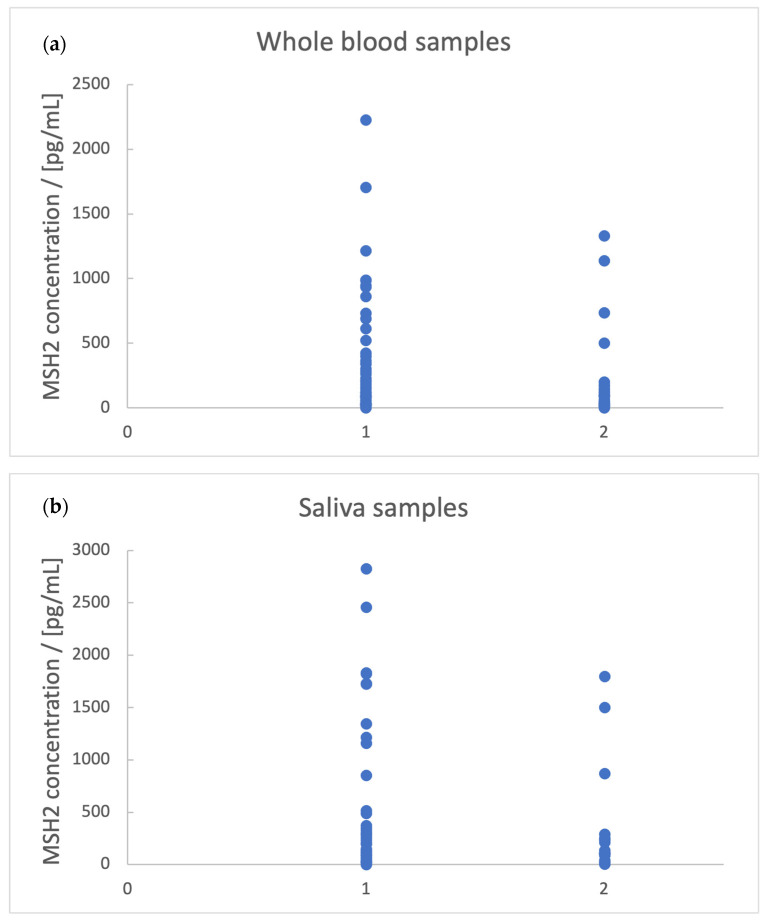
The association between mucus presence and lower values of MSH2 concentration in whole blood (**a**) and saliva samples (**b**). 1—the absence of mucus, 2—the presence of mucus.

The presence of mucus in the tumor mass is slightly related to saliva MSH2 level (*p* = 0.15) and whole blood MSH2 levels (*p* = 0.183), as it was observed that the presence of mucus is associated with lower values of MSH2 concentration. This aspect can be observed in Figure 1.

**Figure 2:**
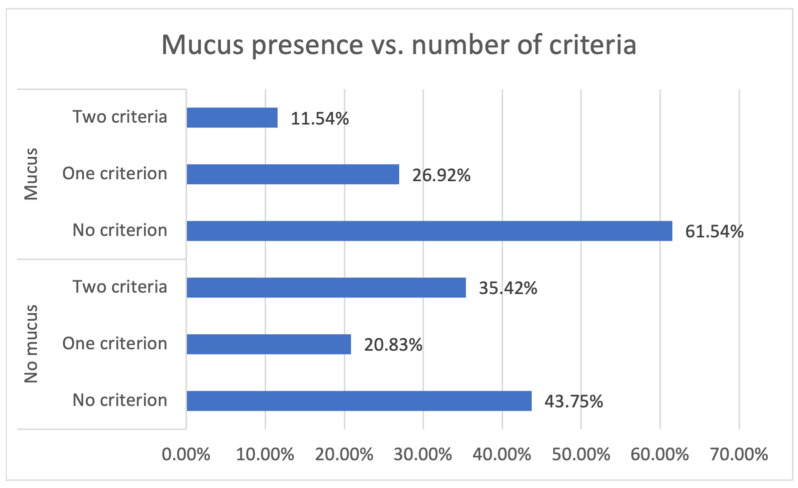
The relationship between mucus absence in the tumor mass and the number of met criteria.

As observed in Figure 1, the cut-off values for MSH2 concentrations can be chosen because only a few patients with MSH2 concentration in whole blood samples > 125 pg/mL and in saliva samples > 140 pg/mL present a mucus compound. By setting these as criteria for the absence of mucus, a correlation between the number of met criteria and the presence of mucus (*p* = 0.044) (Figure 2) was obtained. For the absence and the presence of mucus by the number of criteria, the percentages of patients are 43.75% and 61.54% (for no criterion), 20.83% and 26.92% (for one criterion), and 35.42% and 11.54% (for both criteria). It can be observed that 88.46% of the patients with mucus compounds have at most one criterion and most of them have no criterion. The differentiation between the patients whose tumor mass is associated with mucus presence and the ones whose tumor mass is not associated with mucus presence cannot be made only based on MSH2 levels. This differentiation would require other biomarkers’ levels.

On the other hand, mucinous adenocarcinoma can be excluded and differentiated from other adenocarcinomas using the observation that 71.42% of them are related to MSH2 concentrations < 125 pg/mL and all of them are related to concentrations < 250 pg/mL in saliva samples and, respectively, 87.5% of them are related to concentrations < 100 pg/mL in whole blood samples.

**Figure 3:**
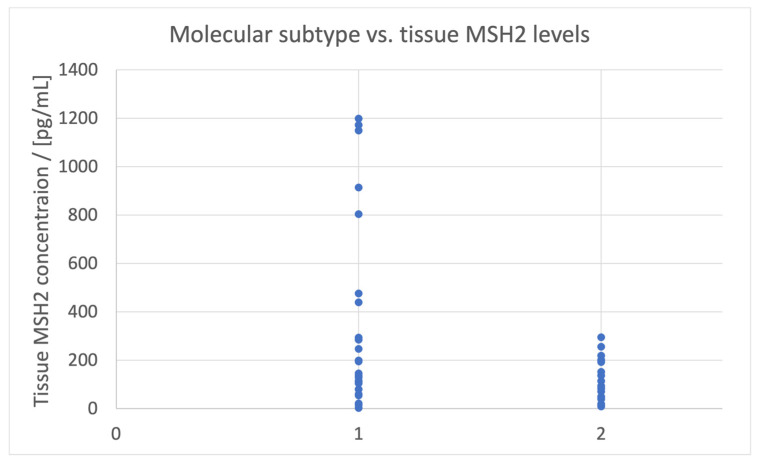
Molecular subtype vs. the levels of MSH2 in tumor tissue samples. 1—epithelial, 2—hybrid or mesenchymal.

The molecular subtype (epithelial, hybrid, or mesenchymal) is related to tissue sample MSH2 concentrations (*p* = 0.017), as higher values are linked to the epithelial subtype (Figure 3).

**Figure 4:**
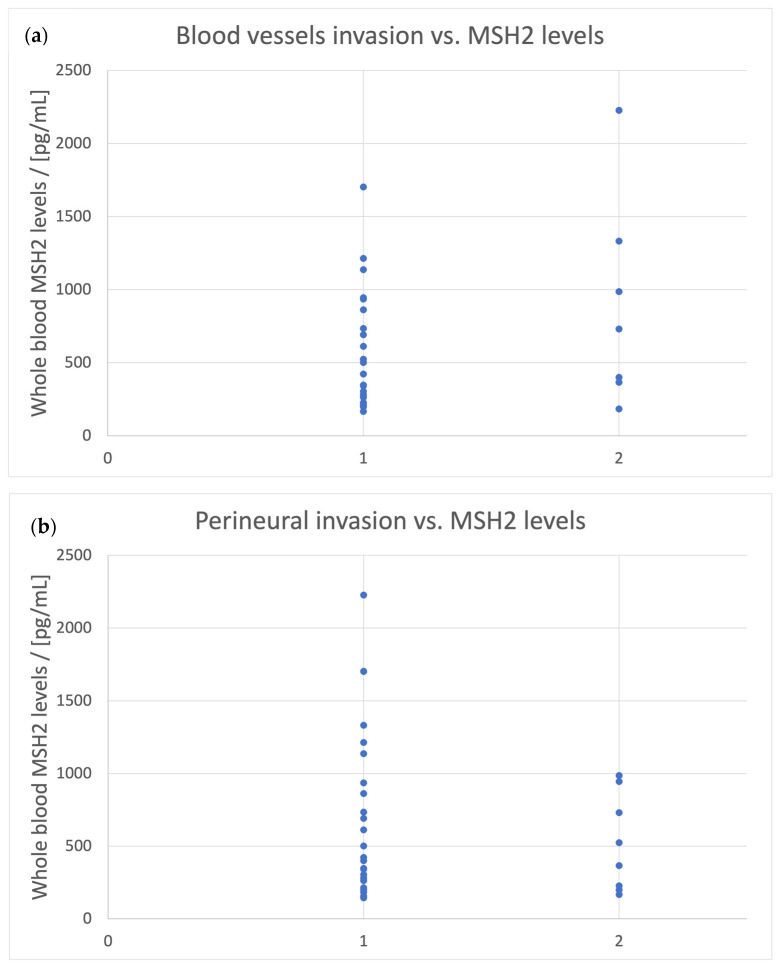
Blood vessels invasion (**a**) and perineural invasion (**b**) vs. the levels of MSH2 in whole blood samples. 1—no invasion, 2—the presence of invasion.

Regarding invasions, some observations were relatively made for whole blood MSH2 levels. For blood vessel invasion, observations can only be made for an MSH2 concentration > 160 pg/mL in whole blood samples. Among those patients, only 22.58% have blood vessel invasion, while the rest of them do not (Figure 4a). A similar observation can be made for perineural invasion, but for concentrations > 130 pg/mL, the percent of the patients with perineural invasion is 23.53% in this case (Figure 4b). 

**Figure 5:**
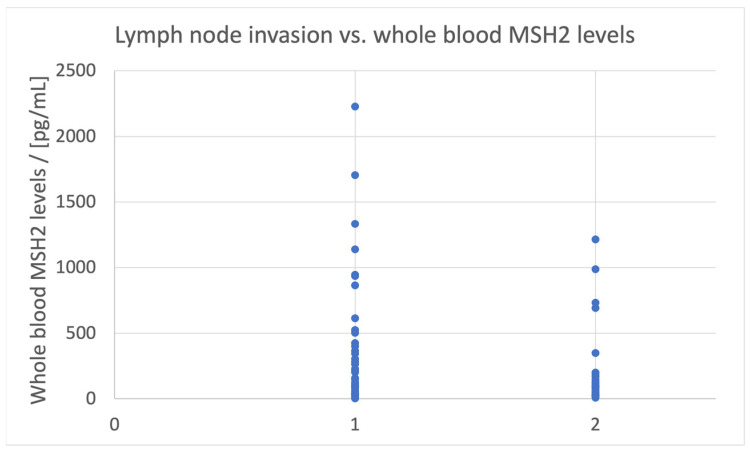
Lymph node invasion vs. the levels of MSH2 in whole blood samples. 1—no invasion, 2—the presence of invasion.

Lymph node invasion was correlated with lower values of MSH2 concentration in whole blood samples (*p* = 0.038), as 80.85% of the patients with lymph node invasion had an MSH2 concentration < 125 pg/mL (Figure 5).

**Figure 6:**
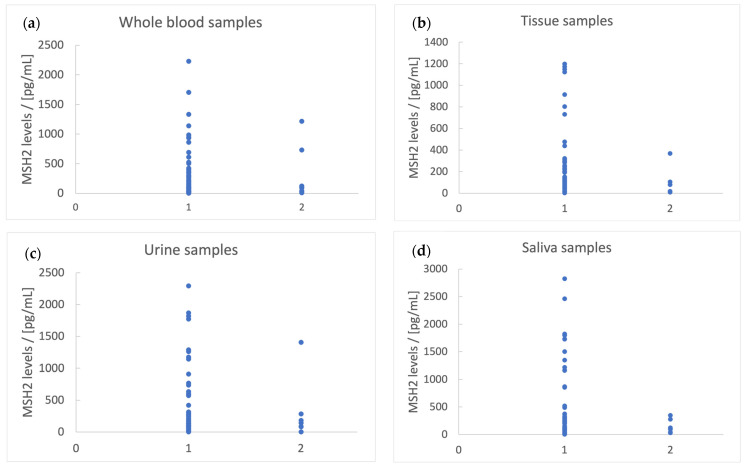
Tumor deposit presence vs. the level of MSH2 in different biological fluids: (**a**) whole blood, (**b**) tissue, (**c**) urine, and (**d**) saliva. 1—lack of tumor deposits, 2—the presence of tumor deposits.

The presence of tumor deposits was slightly correlated with the saliva sample MSH2 level (*p* = 0.23), as it was observed that 75% of the patients with deposits had concentrations < 125 pg/mL, and all of them had concentrations < 350 pg/mL. Similar observations were made for whole blood samples, where 81.8% of the patients with deposits had an MSH2 level < 125 pg/mL, in tissue samples, where 80% of the patients with deposits had an MSH2 level < 125 pg/mL, and in urine samples, where 57.14% of the patients with deposits had an MSH2 level < 150 pg/mL. These can be used at most for exclusion only, as there were many patients with these MSH2 levels who did not have tumor deposits (Figure 6).

### 4.2. MSH6 and Its Clinicopathological Features

The concentration in different biological fluids was determined using stochastic methods, and the results allowed a large series of correlations for determining some important clinicopathological features, as discussed below.

Regarding location, criteria were chosen to reflect the dynamics of the MSH6 molecule in the human body. They are represented by the whole blood MSH6 level, reflecting the mass transfer from tumor to blood; the blood-to-urine ratio (r_1_), reflecting elimination via the kidneys; the blood-to-saliva ratio (r_2_), reflecting elimination via the salivary glands; and the sum S = 2 × [MSH6]_urine_ + [MSH6]_saliva_, reflecting the total elimination in the hypothesis of a urinary output twice the salivary output and, for some locations, the MSH6 level in urine. The criteria were used to evaluate the probability of a location and for the exclusion of a location, as described below. 

The first location to analyze is the rectosigmoid junction and adjacent colorectum area. For this location, the criteria proposed are [MSH6]_whole blood_ > 130 pg/mL, r_1_ < 0.5, r_2_ < 0.83, and S > 1500 pg/mL. All patients with a C5-located tumor met at least three out of the four criteria compared with 14.28% of the C1 patients who had at most three criteria, 40% of the C2 and C3 patients, 6.25% of the C4 patients, and 25% of the C6 patients. These criteria can also be used for the exclusion of C5 if at most two out of the four criteria are met.

After excluding the C5 location, the next location to analyze is C2 and C3. For these two locations, the criteria are [MSH6]_whole blood_ between 130 and 300 pg/mL, r_2_ between 0.3 and 1.2, S > 600 pg/mL, and [MSH6]_urine_ > 250 pg/mL. It was observed that 80% of the patients with C2 or C3 tumors have at least three out of the four criteria compared with no C1 patients, 25% of the patients with a C4 tumor, 50% of the C5 patients, and 35% of the C6 patients. The differentiation between C5, C2, and C3 can be made using the criteria for C5, which are described above. The differentiation from C6 can be made using the MSH6 level in whole blood and urine criteria, as 60% of the patients with C2 or C3 met both criteria compared with only 10% of the C6 patients. The differentiation between C2 and C3 can be made using r_1_. All C2 patients have r_1_ < 0.5, while all C3 patients have r_1_ > 0.5.

The differentiation between C1, C4, and C6 locations cannot be made using MSH6 levels; therefore, it requires other biomarker levels to distinguish.

Regarding gross features, the tumor masses were classified as vegetant, ulcero-vegetant, ulcero-infiltrative, and infiltrative. This classification was slightly associated with urine levels (*p* = 0.134), as it is observed that for the patients with [MSH6]_urine_ > 220 pg/mL, 57.9% are infilitrative or ulcero-infiltrative, 26.31% are ulcero-vegetant and only 15.79% are vegetant. 

The presence of a mucinous compound is related to tissular MSH6 (*p* = 0.074) and to saliva MSH6 (*p* = 0.059) levels. It was observed that the presence of mucus is associated with higher MSH6 levels in both the tissue and saliva samples. By analyzing the values in the tissue samples, it was observed that 60% of the patients with a mucus compound have MSH6 levels over 250 pg/mL compared with only 33.3% of the patients who do not. On the other hand, by analyzing saliva MSH6 levels and setting the same cut-off value of 250 pg/mL, it was observed that 80% of the patients with mucinous adenocarcinoma have MSH6 levels over 250 pg/mL compared with 54.5% of the patients who have a mucus compound and 36.54% of the patients with no mucus compound.

The molecular subtype (epithelial, mesenchymal, or hybrid) is related to urine MSH6 levels (*p* = 0.044), as higher levels are associated with the presence of a mesenchymal compound. It was observed that 70.27% of the patients with the pure epithelial molecular subtype have levels < 200 pg/mL compared with only 33.3% of the patients with a mesenchymal compound.

Stroma features are related to urine MSH6 levels (*p* = 0.000224), as lower values are linked to inflammatory compounds and higher values to fibrous compounds. It was observed that 92.3% of the patients with inflammatory stroma have MSH6 levels under 250 pg/mL compared with only 42.86% of the patients with mixed inflammatory and fibrous stroma and zero patients with hyalinized stroma. All patients with hyalinized stroma had values over 300 pg/mL.

Regarding invasions, the only invasion linked to MSH6 was a vascular invasion. This is related to urine MSH6 levels (*p* = 0.052), as 94.12% of the patients with an MSH6 level over 450 pg/mL had no vascular invasion.

**Figure 7:**
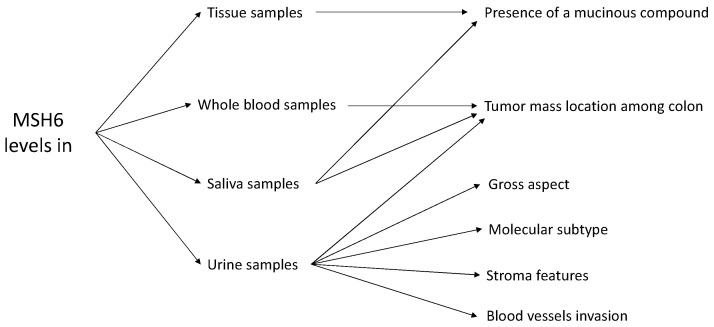
Correlations between MSH6 levels in different biological fluids and clinicopathological features.

All of the above given data are summarized in Figure 7 and in Table 2 in the Section 3.

### 4.3. The Practical Use of the Developed Criteria Using Coding

As all the criteria developed and detailed above were mathematical and empirically established, a code based on them can be developed. The proposed code is given in the Supplementary Materials, and it contains all the necessary practice data from the discussion above. 

The code is based only on the correlations for MSH2 and MSH6. These biomarkers can be used for an approximation with good probability of a series of clinicopathological features. In order to obtain a higher probability, other biomarkers should be used.

## 5. Conclusions

The correlation between the concentrations of MSH2 in the four types of samples and the pathological features allow for a fast characterization of a tumor, which can help a surgeon and an oncologist choose personalized treatments. Also, the colorectal tumor location was correlated with the concentration of MSH2 in whole blood, urine, and saliva.

MSH6, which stands for mutS homolog 6, is not only useful in immunohistochemistry in pathology practice. Its levels can also be correlated with a series of pathological features. In this paper, the relationship between MSH6 levels in four biological fluids—whole blood, saliva, urine, and tissues—and survival rates, the tumor location among the colorectal area, gross features, the presence of a mucinous compounds, molecular subtypes, stroma features, and vascular invasions were presented. 

All this information can be known for a long time before the pathological result as it requires only an obtained sample and an analysis using stochastic methods. This way, in only a couple of hours, an oncologist and a surgeon can have an orientative idea about a tumor, which can be extremely useful for patient management. 

## Figures and Tables

**Table 1:** Cut-off values for clinicopathological features related to MSH2.

Feature	Sample/Parameter	Level (pg/mL)/Value of Parameter
Location	r_1_	1.5
	S	500
	r_2_	0.75
	Whole blood	50
Vegetant grossing aspect	Whole blood	150
Presence of mucus	Whole blood	125
	Saliva	140
Blood vessel invasion	Whole blood	160
Lymph vessel invasion	Whole blood	125
Tumor deposit presence	Saliva	125
	Whole blood	125
	Urine	150

**Table 2:** Cut-off values for clinicopathological features related to MSH6.

Feature	Sample/Parameter	Level (pg/mL)/Value of Parameter
Location—C1 + C4 + C6	r_1_	0.5
	S	1500
	r_2_	0.83
	Whole blood	130
Location—C2 + C3	Urine	250
	S	600
	r_2_	0.3–1.2
	Whole blood	130–300
Location—C5	r_1_	0.5
	S	1500
	r_2_	0.83
	Whole blood	130
Vegetant and ulcero-vegetant grossing aspect	Urine	220
Presence of mucus	Tissue	250
	Saliva	250
Molecular subtype	Urine	200
Stroma features	Urine	250
	Urine	300
Blood vessel invasion	Urine	450

**Table 3:** The location related to the four parameters. The notations used in this table are C_1_ for the ascending colon, C_2_ for the transverse colon, C_3_ for the descending colon, C_4_ for the sigmoid colon, C_5_ for the rectosigmoid junction, and C_6_ for the rectum.

Criteria	[MSH2]_whole blood_ < 50 pg/mL	r_1_ > 1.5	r_2_ > 0.75	S > 500 pg/mL
High probability	C5	C1	C2, C3, C6	C1, C2, C3
Medium probability	C2, C3, C4, C6	-	C1, C4	C4, C6
Low probability	C1	C2, C3, C4, C5, C6	C5	C5

## Data Availability

Data may be available from the corresponding author, subject to reasonable requests, and the approval of the scientific committee of the PN-III-P4-ID-PCCF-2016-0006 project.

## Supplementary Materials

The following supporting information can be downloaded at: https://www.mdpi.com/article/10.3390/biomedicines11123213/s1.
