# Supplementary material for: Correlations between MSH2 and MSH6 Concentrations in Different Biological Fluids and Clinicopathological Features in Colorectal Adenocarcinoma Patients and Their Contribution to Fast and Early Diagnosis of Colorectal Adenocarcinoma
Source: Biomedicines. 2023 Dec 4;11(12):3213. doi: 10.3390/biomedicines11123213 (PMC10741075; doi:10.3390/biomedicines11123213)
Supplement: Supplementary file 1 [file biomedicines-11-03213-s001.zip › biomedicines-2711862-supplementary.pdf]

```
%as the criteria and proposed algorithms are mathematically obtained
%all the parameters discussed above can be obtained
%by using coding

%firstly the levels of MSH2 and MSH6 in different biological fluids have to
%be introduced and further the specific parameters (r1, S and r2) have to
%be calculated

%for MSH2
%in whole blood
MSH2_wb=input('The level of MSH2 in whole blood samples (pg/mL) is ');

%in urine
MSH2_urine=input('The level of MSH2 in urine samples (pg/mL) is ');

%in saliva
MSH2_saliva=input('The level of MSH2 in saliva samples (pg/mL) is ');

%in tumor tissue
MSH2_tissue=input('The level of MSH2 in tissue samples (pg/mL) is ');

%the calculus of r1
r1_MSH2=MSH2_wb/MSH2_urine;

%the calculus of r2
r2_MSH2=MSH2_wb/MSH2_saliva;

%the calculus of S
S_MSH2=MSH2_saliva+2*MSH2_urine;

%for MSH6
%in whole blood
MSH6_wb=input('The level of MSH6 in whole blood samples (pg/mL) is ');

%in urine
MSH6_urine=input('The level of MSH6 in urine samples (pg/mL) is ');

%in saliva
MSH6_saliva=input('The level of MSH6 in saliva samples (pg/mL) is ');

%in tumor tissue
MSH6_tissue=input('The level of MSH6 in tissue samples (pg/mL) is ');

%the calculus of r1
r1_MSH6=MSH6_wb/MSH6_urine;

%the calculus of r2
r2_MSH6=MSH6_wb/MSH6_saliva;

%the calculus of S
S_MSH6=MSH6_saliva+2*MSH6_urine;

%the first feature discussed is the location
```

```
%there will be defined two parameters in order to appreciate the location
%the checking parameter
x_ck_MSH6=0;
x_ck_MSH2=0;

%the number of criteria parameter
n_cr=0;

%firstly, there will be used MSH6 levels to anticipate the location

if r1_MSH6 <0.5
   n_cr=n_cr+1;
end

if S_MSH6>1500
   n_cr=n_cr+1;
end

if r2_MSH6<0.83
   n_cr=n_cr+1;
end

if MSH6_wb>130
   n_cr=n_cr+1;
end

if n_cr>2
   display('By using MSH6 criteria, the location of tumor is probably in the rectosigmoid juction
area')
   x_ck_MSH6=1;
end

n_cr=0;

if x_ck_MSH6==0;
   if MSH6_urine >250
   n_cr=n_cr+1;
   end

   if S_MSH6>600
   n_cr=n_cr+1;
   end

   if r2_MSH6<1.2
      if r2_MSH6>0.3
      n_cr=n_cr+1;
      end
   end

   if MSH6_wb>130
      if MSH6_wb<300
      n_cr=n_cr+1;
      end
   end
```

```
        if n_cr>2
            if r1_MSH6<0.5
        display('By using MSH6 criteria, the location of tumor is probably in the transverse colon
area')
            else
        display('By using MSH6 criteria, the location of tumor is probably in the descendent colon
area')
            end
        x_ck_MSH6=1;
        end
    end

    %the levels of MSH2 will be used further to anticipate the location

    n_cr=0;

    if r1_MSH2 >1.5
        n_cr=n_cr+1;
    end

    if S_MSH2>1500
        n_cr=n_cr+1;
    end

    if r2_MSH2<0.75
        n_cr=n_cr+1;
    end

    if MSH2_wb>50
        n_cr=n_cr+1;
    end

    if n_cr>2
        display('By using MSH2 criteria, the location of tumor is probably in the ascendent colon area')
        x_ck_MSH2=1;
    end

    if x_ck_MSH6==0
        if x_ck_MSH2==0;
                display('By using MSH2 and MSH6 criteria for excluding the other options, the location of
tumor is probably in the sigmoid colon or rectum area')
        end
    end

    %the next feature discussed is the macroscopic grossing appearance feature

    if MSH2_wb<150
        display('By using MSH2 criteria, the tumor probably has a vegetant macroscopic compound')
    else
        display('By using MSH2 criteria, the tumor probably has no vegetant macroscopic compound')
    end

    if MSH6_urine<150
```

```
        display('By using MSH6 criteria, the tumor probably has a vegetant or ulcero-vegetant
macroscopic compound')
    else
        display('By using MSH6 criteria, the tumor probably has no vegetant macroscopic compound')
    end

    %the presence of a mucus compound will be evaluated further

    n_cr=0;

    if MSH2_wb<125
        n_cr=n_cr+1;
    end

    if MSH2_saliva<140
        n_cr=n_cr+1;
    end

    if n_cr>0
        display('By using MSH2 criteria, the tumor probably has a mucus compound')
    else
        display('By using MSH2 criteria, the tumor probably has no mucus compound')
    end

    n_cr=0;

    if MSH6_tissue>250
        n_cr=n_cr+1;
    end

    if MSH6_saliva>250
        n_cr=n_cr+1;
    end

    if n_cr>0
        display('By using MSH6 criteria, the tumor probably has a mucus compound')
    else
        display('By using MSH6 criteria, the tumor probably has no mucus compound')
    end


    %the molecular subtype is related to urinary MSH6 level

    if MSH6_urine<200
        display('By using MSH6 criteria, the tumor probably has an epithelial compound')
    end

    %stroma features features are related to urinary MSH6 levels too

    if MSH6_urine<250
        display('By using MSH6 criteria, the tumor stroma is probably inflammatory or mixed, but
with higher inflammatory compound')
    else
    if MSH6_urine>300
```

```
    display('By using MSH6 criteria, the tumor stroma is probably fibrous even hyalinized')
else
    display('By using MSH6 criteria, the tumor stroma is probably mixed')
end
end

%the next discussion is about invasions as blood vessels and lymph vessels
%invasions are related to MSH2 and MSH6 levels

%for blood vessels invasion

if MSH2_wb<160
    display('By using MSH2 criteria, the tumor probably has blood vessels invasion')
    else
    display('By using MSH2 criteria, the tumor probably has no blood vessels invasion')
end

if MSH6_urine>450
    display('By using MSH6 criteria, the tumor probably has no blood vessels invasion')
else
    display('By using MSH6 criteria, the tumor probably has no blood vessels invasion')
end

% for lymph vessels invasion

if MSH2_wb<125
    display('By using MSH2 criteria, the tumor probably has lymph vessels invasion')
else
    display('By using MSH2 criteria, the tumor probably has no lymph vessels invasion')
end

%the last feature presented in this paper is tumor deposits presence

n_cr=0;

if MSH6_saliva<125
    n_cr=n_cr+1;
end

if MSH6_wb<125
    n_cr=n_cr+1;
end

if MSH6_urine<150
    n_cr=n_cr+1;
end

if n_cr>1
    display('By using MSH2 criteria, the tumor probably associates tumor deposits')
else
    display('By using MSH2 criteria, the tumor probably does not associate tumor deposits')
end
```
